# On solving system of differential-algebraic equations using adomian decomposition method

**DOI:** 10.12688/f1000research.140257.1

**Published:** 2023-10-16

**Authors:** Srinivasarao Thota, Shanmugasundaram P

**Affiliations:** 1 Department of Mathematics, Amrita Vishwa Vidyapeetham, Amaravati, Andhra Pradesh, 522503, India; 2 Department of Mathematics, Mizan-Tepi University, Mizan Teferi, Southern Nations, Nationalities, and People's Region, Ethiopia

**Keywords:** Differential-algebraic equations, Adomian decomposition method, Approximate solutions.

## Abstract

**Background:** In this paper, we focus on an efficient and easy method for solving the given system of differential-algebraic equations (DAEs) of second order.

**Methods:** The approximate solutions are computed rapidly and efficiently with the help of a semi-analytical method known as Adomian decomposition method (ADM). The logic of this method is simple and straightforward to understand.

**Results:** To demonstrate the proposed method, we presented several examples and the computations are compared with the exact solutions to show the efficient. One can employ this logic to different mathematical software tools such as Maple, SCILab, Mathematica, NCAlgebra, Matlab etc. for the problems in real life applications.

**Conclusions:** In this paper, we offered a method for solving the given system of secondorder nonlinear DAEs with aid of the ADM. We shown that the proposed method is simple and efficient, also one can obtain the approximate solutions quickly using this method. A couple of examples are discussed for illustrating this method and graphical and mathematical assessments are discussed with the analytical solutions of the given problems.

## Introduction

The applications of system of differential-algebraic equations (DAEs) occur in many branches of engineering, scientific and real life applications. For example, these equations arise in circuit analysis, electrical networks, computer aided design (CAD), optimal control, real-time simulation of mechanical (multi-body) systems, incompressible fluids dynamics, power system and chemical process simulations. DAEs are a combination of algebraic equations and differential operations, and many mathematical models in different fields are expressed in terms of DAEs. The system of DAEs is a combination of algebraic and differential equations. In the recent years, several algorithms or methods are introduced by various researchers, engineers and scientists to solve the linear/nonlinear system of DAEs and many of them are focused on the numerical solution. ^7^^,^^13^^,^^14^ In the literature, there are many numerical methods available and these are developed using various existing classical methods. For example, in the literature, there are numerical methods with help of *Padé approximation method,* ^4^^,^^5^ there are methods created using *implicit Runge-Kutta methods,* ^36^ also there are methods developed using back difference formula (BDF) ^3^^,^^13^^,^^35^ and etc. Many existing methods are working for low indexed problems or functions. However, using these methods, many real life applications can be solved. There are many other algorithms or methods for solving DAEs and also for differential equations available in the literature. ^20^^–^^34^ In this paper, we propose a general numerical method to solve the second-order system of DAEs using Adomian decomposition method (ADM). There are some general approaches methods available in the literature, ^18^^,^^19^^,^^37^^,^^38^ and these are developed for solving the first order DAEs.

The main aim of this manuscript is to develop a method that gives us quick approximate solutions of a given system of second order DAEs. In order to develop the proposed method, we use a powerful technique, namely ADM, to get the solution of DAEs system. Since 1980, the ADM has been used widely to solve the nonlinear or linear problems in various fields. For example, recently, ADM is widely used as a straightforward powerful tool for solving a large class of nonlinear equations ^1^^,^^2^^,^^8^^–^^12^^,^^15^ such as functional equations, integro-differential equations (IDEs), partial differential equations (PDEs), algebraic equations, differential equations (DEs), differential-delay equations and different kind of equations arise in chemical reactions, physics and biology. We use the ADM to obtain a rapid approximation solution of a given DAEs systems.

This paper is planned as follows: in the next section we recall the ADM to solve the ODEs. The method proposed in this paper for DAEs systems is presented in the following section. Then a number of numerical examples are presented to illustrate the method, followed by concluding remarks.

## Adomian Decomposition Method: An Overview

In this section, we recall ADM briefly to solve ODEs. More details about the ADM can be found in. ^2^^,^^9^^,^^15^^,^^17^ Consider the nonlinear DE of the following type

$$\mathrm{Ly}+\mathrm{Ry}+N\left(y\right)=f,$$ (1)

where L is an non-singular linear operator with the largest-order derivative in the DE, the operator R is the combination of the rest of derivatives in the DE, f is an analytical forcing function and $N\left(y\right)$ is the nonlinear term.

We can solve (1) for y by applying the inverse operator $L^{-1}$ . Indeed, we have the following solution by solving (1) for Ly and then apply the inverse operator $L^{-1}$ on to both sides,

$$\;L^{-1}\mathrm{Ly}=L^{-1}f-L^{-1}\mathrm{Ry}-L^{-1}N\left(y\right)\;\text{or}$$ (2)

$$\;y=g+L^{-1}f-L^{-1}\mathrm{Ry}-L^{-1}N\left(y\right),$$ (3)

where g is depending on the degree of differential operator and initial conditions. In particular, if $\mathrm{Ly}=y^{\prime }=\frac{\mathrm{dy}}{\mathrm{dx}}$ and the initial condition $y\left(0\right)=c_{0}$ , then $L^{-1}=\int_{0}^{x}\cdot \;\mathrm{dx}$ and $L^{-1}\mathrm{Ly}=y-c_{0}$ . In this case $g=c_{0}$ . If $\mathrm{Ly}=y^{\prime \prime }=\frac{d^{2}y}{{\mathrm{dx}}^{2}}$ and the initial condition $y\left(0\right)=c_{0}$ and $y^{\prime }\left(0\right)=c_{1}$ , then $L^{-1}=\int_{0}^{x}\int_{0}^{x}\cdot \;\mathrm{dx}\;\mathrm{dx}$ and $L^{-1}\mathrm{Ly}=y-c_{0}-c_{1}\left(x-0\right)$ . In this case $g=c_{0}+c_{1}x$ .

To apply the ADM to (3), let y be the solution of (1), and it can be expressed in the form of infinite series as follows,

$$y=\sum_{n=0}^{\infty }y_{n},$$ (4)

where the required components of solution $y_{n}$ , n=0,1,2,… can be computed using the ADM. The term $N\left(y\right)$ can be expressed in terms of the Adomian polynomials $N_{n}$ , see for examples, ^10^^–^^12^^,^^36^ as

$$N\left(y\right)=\sum_{n=0}^{\infty }N_{n}\left(y_{0},y_{1},\ldots ,y_{n}\right).$$ (5)

Now, choose $y_{0}$ as

$$y_{0}=g+L^{-1}f,$$ (6)

and rewrite the equation (3) using the equations (4) and (5), we obtain

$$\sum_{n=0}^{\infty }y_{n}=y_{0}-L^{-1}R\sum_{n=0}^{\infty }y_{n}-L^{-1}\sum_{n=0}^{\infty }N_{n}.$$ (7)

On comparing the general terms of (7), we obtain the following equation for the ADM

$$y_{n}=-L^{-1}{\mathrm{Ru}}_{n-1}-L^{-1}N_{n-1},\;n\geq 1.$$ (8)

We have $y_{0}$ from (6), and using (8) we can generate the components $y_{n}$ for an approximate solution. Further, we can obtain the exact solution of (1) if the series (4) converges. The K -order approximation solution is obtained as

$$y\left(t\right)=\sum_{n=0}^{K}y_{n}.$$ (9)

The next section presents a method for DAEs systems using the ADM.

## Proposed Method using ADM

Consider a system of second-order DEs as follows

$$\begin{array}{ll} & y_{1}^{\prime \prime }=f_{1},\\ & y_{2}^{\prime \prime }=f_{2},\\ & \vdots \\ & y_{n}^{\prime \prime }=f_{n}, \end{array}$$ (10)

where $y_{i}^{\prime \prime }$ is the second order derivative of $y_{i}$ respected to the independent variable x , and $f_{1},f_{2},\ldots ,f_{n}$ are n unknown functions.

We can rewrite the system (10), as follows:

$${\mathrm{Ly}}_{i}=f_{i},\;i=1,2,\ldots ,n,$$ (11)

where $L=D^{2}=\frac{d^{2}}{{\mathrm{dx}}^{2}}$ is the differential operator, and its inverse operator $D^{-1}=I=\int_{0}^{x}\cdot \;\mathrm{dx}$ . Hence $L^{-1}=I^{2}$ is the secon-order inverse operator. Now we define the integral or inverse operator for the anti-derivative as follows

$$If\left(x\right)={\int_{0}}^{x}f\left(\xi \right)\;\mathrm{d\xi},$$

and we have DIf=f , that is DI=1 . The higher-order of integral operator $I^{n}$ is defined in the simple way, and each $I^{n}f$ must be continuous. In particular,

$$I^{2}f\left(x\right)=\int_{0}^{x}\int_{0}^{x_{1}}f\left(\xi \right)\;\mathrm{d\xi}\;{\mathrm{dx}}_{1}.$$

From replacement lemma, ^16^ we have the following equation. The replacement lemma helps us to convert the double integral into a single integral as given below,

$$I^{2}f\left(x\right)=x\int_{0}^{x}f\left(\xi \right)\;\mathrm{d\xi}-\int_{0}^{x}\mathrm{\xi f}\left(\xi \right)\;\mathrm{d\xi}.$$ (12)

Thus, (12) can be expressed in terms of integral operator I as follows

$$I^{2}f\left(x\right)=xIf-I\mathrm{xf},$$

and in operator notation, we have $I^{2}=xI-Ix$ . One can easily verify that $D^{2}I^{2}=1$ and also $D^{2}\left(xI-Ix\right)=1$ . We call xI−Ix , the *normal form* of the integral operator $I^{2}$ .

Using the inverse operator on (11), we get

$$y_{i}=y_{i}\left(0\right)+y_{i}^{\prime }\left(0\right)x+x\int_{0}^{x}f_{i}\;\mathrm{dx}-\int_{0}^{x}{\mathrm{xf}}_{i}\;\mathrm{dx},\;i=1,2,\ldots ,n.$$ (13)

Applying ADM, we have the solution of (13) in the series sum,

$$y_{i}=\sum_{j=0}^{\infty }f_{i,j},$$ (14)

and the integrand in (13), as the sum of the following series:

$$f_{i}=\sum_{j=0}^{\infty }A_{i,j}\left(f_{i,0},f_{i,1},\ldots ,f_{i,j}\right),$$ (15)

where $A_{i,j}\left(f_{i,0},f_{i,1},\ldots ,f_{i,j}\right)$ are called Adomian polynomials. ^10^^–^^12^^,^^36^ Putting (14) and (15) into (13), we get

$$\begin{array}{ll} \sum_{j=0}^{\infty }f_{i,j}= & \;y_{i}\left(0\right)+y_{i}^{\prime }\left(0\right)x+x\int_{0}^{x}\sum_{j=0}^{\infty }A_{i,j}\left(f_{i,0},f_{i,1},\ldots ,f_{i,j}\right)\;\mathrm{dx}\\ & -\int_{0}^{x}x\sum_{j=0}^{\infty }A_{i,j}\left(f_{i,0},f_{i,1},\ldots ,f_{i,j}\right)\;\mathrm{dx}, \end{array}$$ (16)

from (8) we define, for n=0,1,… ,

$$\begin{array}{ll} & \;f_{i,0}=y_{i}\left(0\right)+y_{i}^{\prime }\left(0\right)x,\\ & \;f_{i,n+1}=x\int_{0}^{x}A_{i,n}\left(f_{i,0},f_{i,1},\ldots ,f_{i,n}\right)\;\mathrm{dx}-\int_{0}^{x}{\mathrm{xA}}_{i,n}\left(f_{i,0},f_{i,1},\ldots ,f_{i,n}\right)\;\mathrm{dx}. \end{array}$$ (17)

Since $f_{i,0}$ are known, we can use $f_{i,n+1}$ to generate the approximate solution components.

## Numerical Examples

Example 1.
*Consider a system of second order DAEs with initial conditions to illustrate the proposed method.* ^39^

$$\begin{array}{ll} & \;y_{1}^{\prime \prime }-xy_{2}^{\prime }=2y_{1}+y_{2},\\ & \;y_{2}=e^{x}, \end{array}$$ (18)

and initial conditions are $y_{1}\left(0\right)=y_{2}\left(0\right)=y_{2}^{\prime }\left(0\right)=1,y_{1}^{\prime }\left(0\right)=0$ . The exact solution of this system is

$$\begin{array}{l} y_{1}=\left(2+\sqrt{2}\right)e^{\sqrt{2}x}+\left(2-\sqrt{2}\right)e^{-\sqrt{2}x}-\left(x+3\right)e^{x},\\ y_{2}=e^{x}. \end{array}$$

*In order to apply the proposed method, we rewrite the given system* (18) *as follows*

$$\;y_{{1^{\prime }}^{\prime }}={\mathrm{xy}}_{2^{\prime }}+2y_{1}+y_{2},$$

$$\;y_{2}=e^{x}.$$

*On simplifying above equations, we have* $y_{1}^{\prime \prime }=2y_{1}+\left(x+1\right)e^{x}$ *. Following procedure as given in* (13) *, we get*

y1=y10+y1′0x+x∫0x2y1+x+1exdx−∫0xx2y1+x+1exdx=1+x∫0x2y1+x+1exdx−∫0x2xy1+x2+xexdx=1+x∫0xx+1exdx−∫0xx2+xexdx+2x∫0xy1dx−2∫0xxy1dx.

*Use the alternate algorithm to find the Adomian polynomials as given in,* ^6^^,^^10^^–^^12^ *the Adomian method is as following:*

y1,0=1+x∫0xx+1exdx−∫0xx2+xexdx=2+xex−ex,y1,n+1=2x∫0xy1,ndx−2∫0xxy1,ndx.

*We have iterations (approximate solutions components) from above equations as follows*

y1,0=2+xex−ex,y1,1=2xex+2x2−6ex+4x+6,y1,2=16x+4xex+13x4+43x3+6x2+20−20ex,y1,3=48x+163x3+8xex+145x6+215x5+x4+20x2+56−56ex.

*Now we have the approximate solution after three steps*

$$\begin{array}{l} y_{{\mathrm{apx}}_{3}}=\sum_{j=0}^{3}f_{1,j}=84+15{\mathrm{xe}}^{x}-83e^{x}+28x^{2}+68x\\ +\frac{4}{3}x^{4}+\frac{20}{3}x^{3}+\frac{1}{45}x^{6}+\frac{1}{15}x^{5}. \end{array}$$

*After nine steps, we have the solution*

yapx9=17412+16388x+1023xex−17411ex+7684x2+1724324300x13+189100x12+1347351004000x17+110216206000x16+744550x11+199450x10+431890x9+29126x8+1510810300x15+214105x7+70645x6+153815x5+16663x4+16252318072000x18+71723x3+124324300x14.

**Figure 1.**
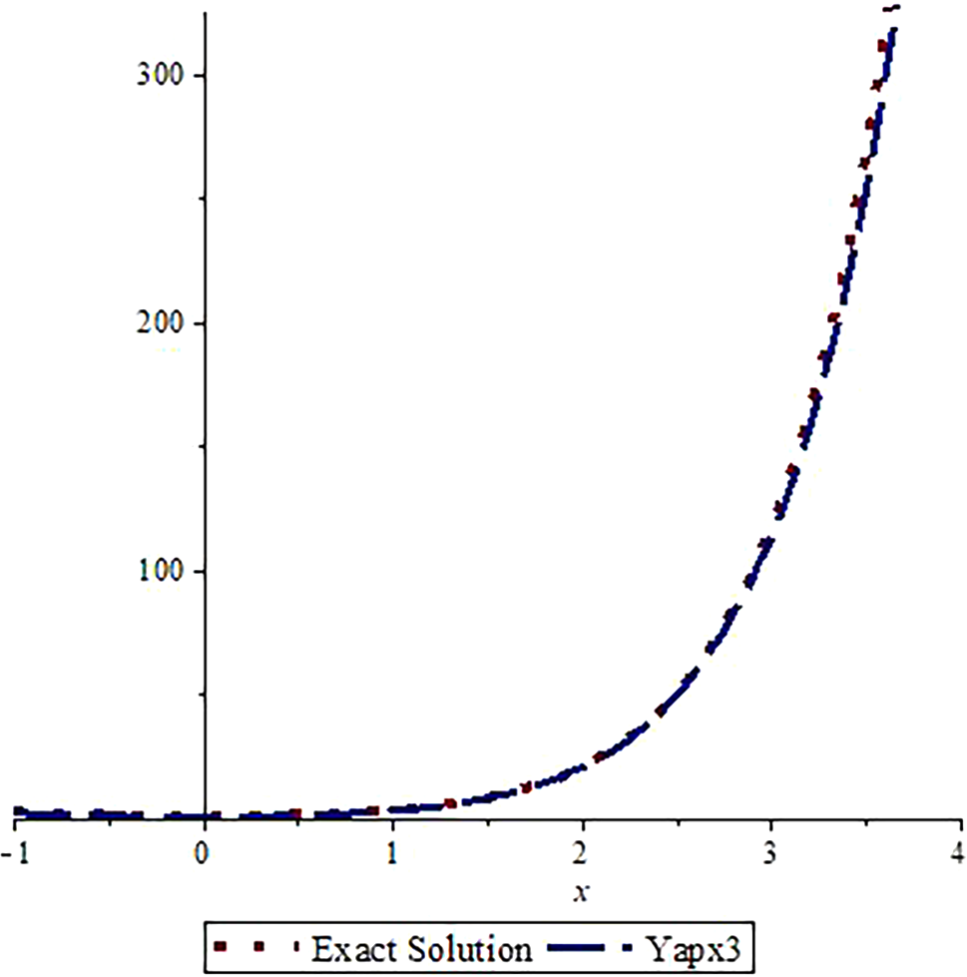
Assessment of $y_{{\mathrm{apx}}_{3}}$ with Exact solution $y_{1}$ .

**Figure 2.**
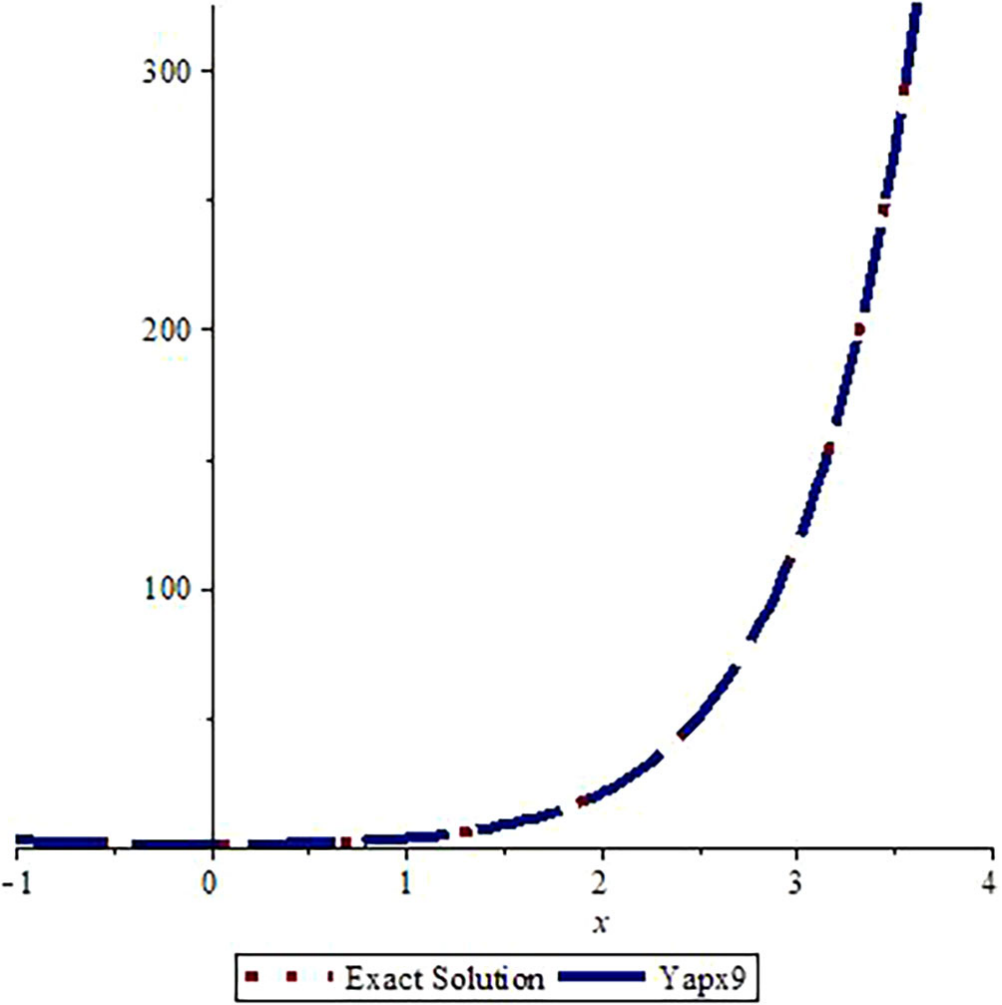
Assessment of $y_{{\mathrm{apx}}_{9}}$ with Exact solution $y_{1}$ .

*Graphical assessment of the analytic solution with the approximate solution after three steps is visualized in* *Figure 1* *and the comparison of the exact solution with approximate solution after nine steps is shown in* *Figure 2*. *From these figures, we can observe that the approximate solutions are near to the analytic solution. A greater number of steps gives us a more accurate solution (the graphs are drawn using Maple 16.0).*

*Numerical results of the exact solution, approximate solution* $y_{{\mathrm{apx}}_{3}}$ *after three steps, approximate solution* $y_{{\mathrm{apx}}_{9}}$ *after nine steps and absolute error are given in* *Table 1*. *From the numerical values in* *Table 1*, *one can observe that the solution* $y_{{\mathrm{apx}}_{9}}$ *is closer to the exact solution* $y_{1}$ *. To get more appropriate solution of the given system, we increase the number of iterations.*

Example 2.
*Consider a DAEs system of second order.* ^39^

$$\begin{array}{ll} & \;y_{1}^{\prime \prime }-2{\mathrm{xy}}_{3^{\prime }}-y_{1}-2y_{2}=0,\\ & \;y_{2}^{\prime \prime }+y_{2}-2y_{3}=2e^{x},\\ & \;y_{3}=\cos x, \end{array}$$ (19)

*with initial conditions* $y_{1}\left(0\right)=0,y_{1}^{\prime }\left(0\right)=y_{2}\left(0\right)=y_{2}^{\prime }\left(0\right)=1$ *. The analytical solution of this system is* $y_{1}={\mathrm{xe}}^{x},y_{2}=e^{x}+x\sin x,y_{3}=\cos x$ *. After simplifying the system* (19) *, we get*

$$\begin{array}{l} \;y_{1}^{\prime \prime }=y_{1}+2y_{2}-2x\sin x,\\ \;y_{2}^{\prime \prime }=-y_{2}+2e^{x}+2\cos x,\\ \;y_{3}=\cos x. \end{array}$$

*Following the procedure of the proposed method, similar to* *Example 1*, *we get*

y1=y10+y1′0x+x∫0xy1+2y2−2xsinxdx−∫0xxy1+2y2−2xsinxdx=x−2x∫0xxsinxdx+2∫0xxsinxdx+x∫0xy1+2y2dx−∫0xy1+2y2dx,y2=y20+y2′0x+x∫0x−y2+2ex+2cosxdx−∫0xx−y2+2ex+2cosxdx=1+x+2x∫0xex+cosxdx−2∫0xxex+cosxdx−x∫0xy2dx+∫0xy2dx.

*Using the alternate algorithm for computing the Adomian polynomials, we have*

y1,0=x−x∫0x2xsinxdx+∫0x2x2sinxdx=x−4+4cosx+2xsinx,y2,0=1+x+2x∫0xex+cosxdx−2∫0xxex+cosxdx=1−x+2ex−2cosx,y1,n+1=x∫0xy1,n+y2,ndx−∫0xy1,n+y2,ndx,y2,n+1=−x∫0xy2,ndx+∫0xy2,ndx.

*Now, we can get iterations from above equations as follows*

y1,0=x−4+4cosx+2xsinx,y2,0=1−x+2ex−2cosx,y1,1=−4x−15x3−x2−4cosx−2xsinx+4ex,y2,1=4+2x−12x2+16x3−2ex−2cosx,y1,2=−12+1120x5−16x4+12cosx+2xsinx+4x2,y2,2=−2x−2x2−13x3+124x4−1120x5+2ex−2cosx,⋮

*After five steps, we have the solution*

y1,apx5=−12−11x+12ex−11008x7−110080x8−1120x6−7120x5−13x4−5x2−1120960x9−32x3−139916800x11−11814400x10,y2,apx5=13+x−12cosx+15040x7+18064x8−1144x6+1120x5+38x4−92x2+1362880x9+16x3+139916800x11−13628800x10.

**Figure 3.**
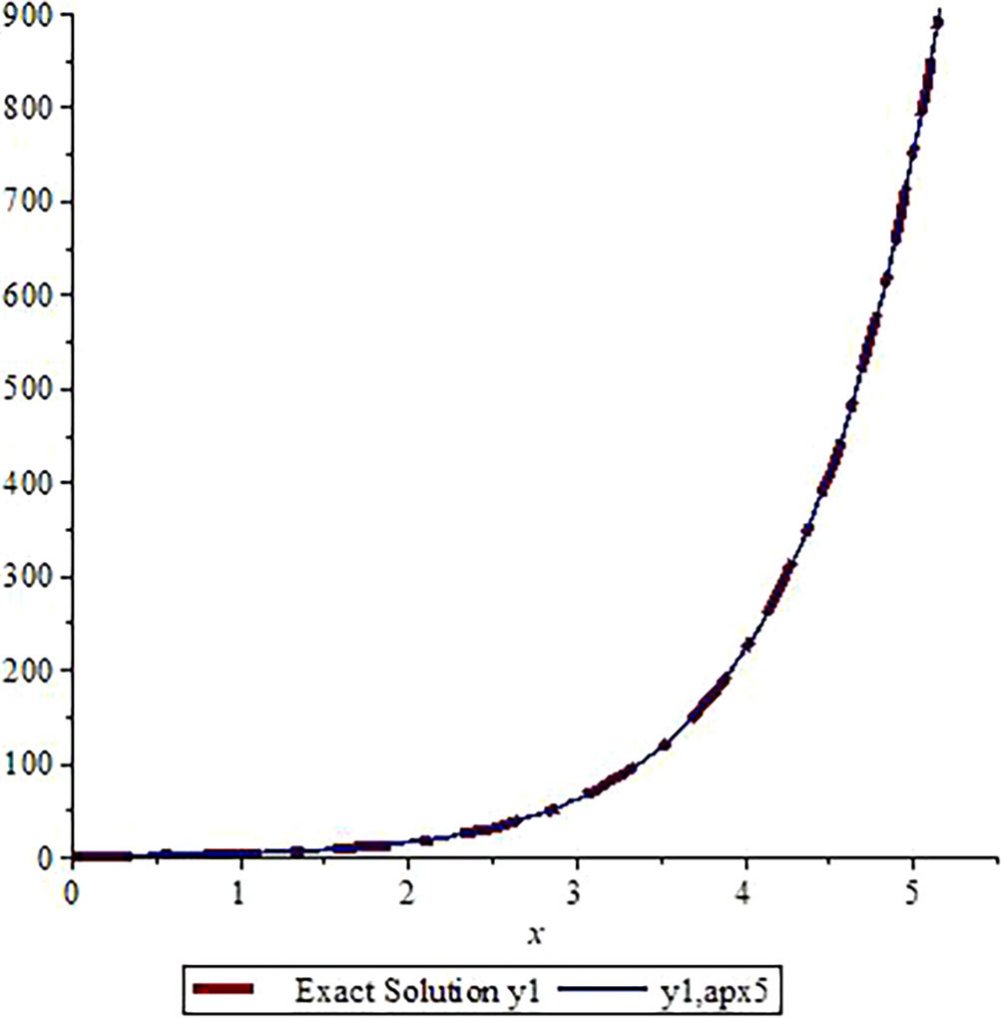
Assessment of $y_{1,{\mathrm{apx}}_{5}}$ with Exact solution $y_{1}$ .

**Figure 4.**
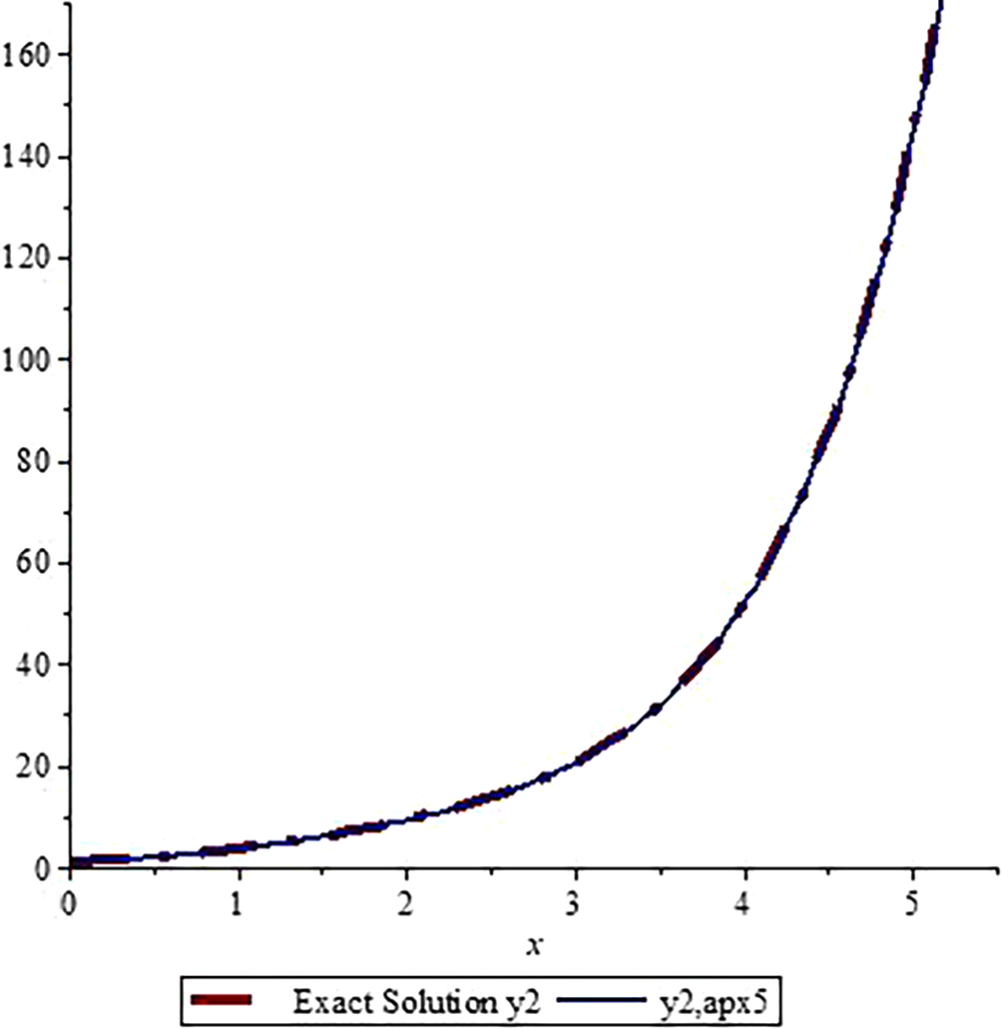
Assessment of $y_{2,{\mathrm{apx}}_{5}}$ with Exact solution $y_{2}$ .

*In* *Figure 3* *and* *Figure 4* *, we show the graphical comparisons of the exact solutions* $y_{1}\left(x\right),y_{2}\left(x\right)$ *with the approximate solution after five steps respectively. From the graphs in* *Figure 3* *and* *Figure 4* *, one can observe that the approximate solutions are very close to the exact solution. Higher number of iterations give us more accurate solution (one can use Microsoft Excel to draw the graphs).*

*In* *Table 2* *and* *Table 3*, *mathematical results of the analytical solution and approximate solutions after five steps* $y_{1,{\mathrm{apx}}_{5}},y_{2,{\mathrm{apx}}_{5}}$ *with absolute errors are given respectively. From these numerical results, one can observe that the approximate solutions* $y_{1,{\mathrm{apx}}_{5}}$ *and* $y_{2,{\mathrm{apx}}_{5}}$ *are closer to the exact solution* $y_{1}$ *and* $y_{2}$ *respectively. For more appropriate solution of the given system, we increase the number of iterations.*

**Table 1.** Mathematical results for Example 1.

x	$y_{1}\left(x\right)$	$y_{{\mathrm{apx}}_{3}}\left(x\right)$	$y_{{\mathrm{apx}}_{9}}\left(x\right)$	$\left|y_{1}\left(x\right)-y_{{\mathrm{apx}}_{9}}\left(x\right)\right|$
−2.0	9.977284737	9.862668553	9.977273565	$1.117\times 10^{-5}$
−1.0	2.503779856	2.503370315	2.503780041	$1.850\times 10^{-7}$
−0.5	1.355157416	1.355155845	1.3551542	$3.216\times 10^{-6}$
0.5	1.442726178	1.442724586	1.4427204	$5.778\times 10^{-6}$
1.0	3.31280240	3.312391255	3.312807041	$4.641\times 10^{-5}$
1.5	8.38448291	8.373436300	8.384502002	$1.909\times 10^{-5}$
2.0	20.85383714	20.73558235	20.85388357	$4.643\times 10^{-5}$
2.5	50.16639112	49.39270508	50.16642510	$3.398\times 10^{-5}$
3.0	117.0950239	113.3495974	117.0950934	$6.950\times 10^{-5}$
3.5	266.6334832	251.7748955	266.6334147	$6.850\times 10^{-5}$
4.0	595.1225779	543.7981055	595.1220909	$4.870\times 10^{-4}$

**Table 2.** Mathematical results for Example 2.

x	Exact value $y_{1}\left(x\right)$	$y_{1,{\mathrm{apx}}_{5}}\left(x\right)$	$\left|y_{1}\left(x\right)-y_{1,{\mathrm{apx}}_{5}}\left(x\right)\right|$
0.1	0.1105170918	0.1105170950	$3.2\times 10^{-9}$
0.2	0.2442805516	0.2442805537	$2.1\times 10^{-9}$
0.4	0.5967298792	0.5967298887	$9.5\times 10^{-9}$
0.6	1.093271280	1.093271277	$3.0\times 10^{-9}$
0.8	1.780432742	1.780432741	$1.0\times 10^{-9}$
1.0	2.718281828	2.718281829	$1.0\times 10^{-9}$

**Table 3.** Mathematical results for Example 2.

x	Exact value $y_{2}\left(x\right)$	$y_{2,{\mathrm{apx}}_{5}}\left(x\right)$	$\left|y_{2}\left(x\right)-y_{2,{\mathrm{apx}}_{5}}\left(x\right)\right|$
0.1	1.115154260	1.115154263	$3.0\times 10^{-9}$
0.2	1.261136624	1.261136629	$5.0\times 10^{-9}$
0.4	1.647592035	1.647592033	$2.0\times 10^{-9}$
0.6	2.160904284	2.160904284	0
0.8	2.799425801	2.799425801	0
1.0	3.559752813	3.559752811	$2.0\times 10^{-9}$

## Conclusions

In this paper, we offered a method for solving the given system of second-order nonlinear DAEs with aid of the ADM. We shown that the proposed method is simple and efficient, also one can obtain the approximate solutions quickly using this method. Logic of the method in this paper is straightforward and simple. The proposed algorithm is focused on second-order DAEs, however we can extend this algorthm to higher-order DAEs systems. One can employ this method to different mathematical software tools such as Mathematica, NCAlgebra, Matlab, Maple, SCILab, *etc.*

## Data Availability

Mendeley data: On solving system of DAEs using ADM https://doi.org/10.17632/r89zy3y657.1. ^39^ This project contains the following underlying data:
•
Paper_Example_1.mw •
Paper_Example_2.mw Data are available under the terms of the Creative Commons Attribution 4.0 International license (CC-BY 4.0).

## References

[1] Wazwaz AM : A new approach to the nonlinear advection problem: An application of the decomposition technique. *Appl. Math. Comput.* 1995;72:175–181. 10.1016/0096-3003(94)00182-4

[2] Benhammouda B : A novel technique to solve nonlinear higher-index Hessenberg differential-algebraic equations by Adomian decomposition method. *Springerplus.* 2016;5:590. 10.1186/s40064-016-2208-3 27330880 PMC4868070

[3] Gear CW Petzold LR : ODE systems for the solution of differential algebraic systems. *SIAM J. Numer. Anal.* 1984;21:716–728. 10.1137/0721048

[4] Çelik E Karaduman E Bayram M : Numerical Method to Solve Chemical Differential-Algebraic Equations. *Int. J. Quantum Chem.* 2002;89:447–451. 10.1002/qua.10305

[5] Çelik E Bayram M : On the numerical solution of differential-algebraic equations by Padé series. *Appl. Math. Comput.* 2003;137:151–160. 10.1016/S0096-3003(02)00093-0

[6] Çelik E Bayram M Yeloğlu T : Solution of Differential-Algebraic Equations (DAEs) by Adomian Decomposition Method. *International Journal Pure & Applied Mathematical Sciences.* 2006;3(1):93–100.

[7] Hairer E Norsett SP Wanner G : *Solving Ordinary Differential Equations II. Stiff and Differential-Algebraic Problems.* New York: Springer;1992. 978-3-642-05221-7.

[8] Aomian G : *Nonlinear Stochastic Systems Theory and Applications to Physics.* Dordrecht/Norwell, MA: Kluwer Acaemic;1989.

[9] Adomian G Rach R : On the solution of algebraic equations by the decomposition method. *J. Math. Anal. Appl.* 1985;105(1):141–166. 10.1016/0022-247X(85)90102-7

[10] Duan JS : Recurrence triangle for Adomian polynomials. *Appl. Math. Comput.* 2010;216:1235–1241. 10.1016/j.amc.2010.02.015

[11] Duan JS : An efficient algorithm for the multivariable Adomian polynomials. *Appl. Math. Comput.* 2010;217:2456–2467. 10.1016/j.amc.2010.07.046

[12] Duan JS : Convenient analytic recurrence algorithms for the Adomian polynomials. *Appl. Math. Comput.* 2011;217:6337–6348. 10.1016/j.amc.2011.01.007

[13] Brenan KE Campbell SL Petzold LR : Numerical Solution of Initial Value Problems in Differential-Algebraic Equations. *SIAM Philadelphia.* 1989;26:976–996. 978-0-89871-353-4. 10.1137/0726054

[14] Petzold LR : Numerical Solution of Differential-Algebraic Equations. *Advances in Numerical Analysis IV.* 1995.

[15] Almazmumy M Hendi FA Bakodah HO : Recent modifications of Adomian decomposition method for initial value problem in ordinary differential equations. *Am. J. Comput. Math.* 2012;02:228–234. 10.4236/ajcm.2012.23030

[16] Peter J : *Collins: Differential and integral equations.* Oxford University Press;2006.

[17] Ramana PV Raghu Prasad BK : Modified Adomian decomposition method for Van der Pol equations. *Int. J. Non Linear Mech.* 2014;65:121–132. 10.1016/j.ijnonlinmec.2014.03.006

[18] Campbell SL : A Computational method for general higher index singular systems of differential equations. *IMACS Trans. Sci. Comput.* 1989;89:555–560.

[19] Campbell SL Moore E Zhong Y : Utilization of automatic differentiation in control algorithms. *IEEE Trans. Automat. Control.* 1994;39:1047–1052. 10.1109/9.284891

[20] Thota S Kumar SD : Solving system of higher-order linear differential equations on the level of operators. *International journal of pure and applied mathematics.* 2016;106(1):11–21. 10.12732/ijpam.v106i1.2

[21] Thota S Kumar SD : On a mixed interpolation with integral conditions at arbitrary nodes. *Cogent Mathematics.* 2016;3(1):1–10. 10.1080/23311835.2016.1151613

[22] Thota S Kumar SD : Symbolic algorithm for a system of differential-algebraic equations. *Kyungpook Mathematical Journal.* 2016;56(4):1141–1160. 10.5666/KMJ.2016.56.4.1141

[23] Thota S Kumar SD : A new method for general solution of system of higher-order linear differential equations. *International Conference on Inter Disciplinary Research in Engineering and Technology.* 2015;1:240–243.

[24] Thota S Kumar SD : Symbolic method for polynomial interpolation with Stieltjes conditions. *International Conference on Frontiers in Mathematics.* 2015;225–228.

[25] Thota S Rosenkranz M Kumar SD : Solving systems of linear differential equations over integro-differential algebras. *International Conference on Applications of Computer Algebra, June 24–29, 2012, Sofia, Bulgaria.*

[26] Thota S : A Study on Symbolic Algorithms for Solving Ordinary and Partial Linear Differential Equations, Ph.D. thesis. 2017.

[27] Thota S Kumar SD : Maple Implementation of Symbolic Methods for Initial Value Problems. *Research for Resurgence-An Edited Multidisciplinary Research Book.* 2017;I:240–243.

[28] Thota S : On a Symbolic Method for Fully Inhomogeneous Boundary Value Problems. *Kyungpook Mathematical Journal.* 2019;59(1):13–22.

[29] Thota S : On A New Symbolic Method for Initial Value Problems for Systems of Higher-order Linear Differential Equations, International Journal of. *Mathematical Models and Methods in Applied Sciences.* 2018;12:194–202.

[30] Thota S : A Symbolic Algorithm for Polynomial Interpolation with Integral Conditions. *Applied Mathematics & Information Sciences.* 2018;12(5):995–1001. 10.18576/amis/120512

[31] Thota S : Initial value problems for system of differential-algebraic equations in Maple. *BMC. Res. Notes.* 2018;11:651. 10.1186/s13104-018-3748-0 30189891 PMC6127927

[32] Thota S : On A Symbolic Method for Error Estimation of a Mixed Interpolation. *Kyungpook Mathematical Journal.* 2018;58(3):453–462.

[33] Thota S : A Symbolic Algorithm for Polynomial Interpolation with Stieltjes Conditions in Maple. *Proceedings of the Institute of Applied mathematics.* 2019;8(2):112–120.

[34] Thota S : On A New Symbolic Method for Solving Two-point Boundary Value Problems with Variable Coefficients, International Journal of. *Math. Comput. Simul.* 2019;13:160–164.

[35] Ascher UM : On symmetric schemes and differential-algebraic equations. *SIAM J. Sci. Stat. Comput.* 1989;10:937–949. 10.1137/0910054

[36] Ascher UM Petzold LR : Projected implicit Runge Kutta methods for differential-algebraic equations. *SIAM J. Numer. Anal.* 1991;28:1097–1120. 10.1137/0728059

[37] Ascher UM Lin P : Sequential regularization methods for higher index differential-algebraic equations with constant singularities: the linear index-2 case. *SIAM J. Numer. Anal.* 1996;33:1921–1940. 10.1137/S0036142993253254

[38] Ascher UM Lin P : Sequential regularization methods for non-linear higher index differential-algebraic equations. *SIAM J. Sci. Comput.* 1997;18:160–181. 10.1137/S1064827595287778

[39] Palanisamy S Thota S : On solving system of DAEs using ADM.[Data]. *Mendeley Data.* 2023;V1. 10.17632/r89zy3y657.1

