# Supplementary material for: On solving system of differential-algebraic equations using adomian decomposition method
Source: F1000Res. 2024 Jan 29;12:1337. Originally published 2023 Oct 16. [Version 2] doi: 10.12688/f1000research.140257.2 (PMC11112463; doi:10.12688/f1000research.140257.2)
Supplement: Peer review history

# Reviewer report: Reviewer response for version 2

**DOI:** 10.5256/f1000research.161819.r243839

**Published:** 2024-05-14

**Authors:** Arun Prasath GM

**Affiliations:** 1 Department of Information Technology, University of Technology and Applied Sciences, Muscat, Oman

This paper focused on an efficient and easy method to solve the second-order systems of differential-algebraic equations (DAEs). They computed the approximate solutions with the help of a semi-analytical method known as “Adomian decomposition method” (ADM). They claimed that the logic of the proposed method is simple and straightforward to understand. Authors presented few examples to illustrate the method. 

 The paper is attractive and planned fine. The key of this paper is to solve the given system of DAEs with the help of ADM to give speedy approximate solution. The design of the work in this paper is constructive and novel. I recommend, this paper can be acceptable.  After review, the authors can include the following comments.

 Comments:

 This paper solves second order DAEs. Can we apply the same logic for higher order DAEs, if so; put it as NOTE point in the conclusion section. 

 Integral operator is fixed with lower-limit as zero or it can be any constant? Give explanation about it.

Is the work clearly and accurately presented and does it cite the current literature?

Yes

If applicable, is the statistical analysis and its interpretation appropriate?

Not applicable

Are all the source data underlying the results available to ensure full reproducibility?

Yes

Is the study design appropriate and is the work technically sound?

Yes

Are the conclusions drawn adequately supported by the results?

Yes

Are sufficient details of methods and analysis provided to allow replication by others?

Yes

Reviewer Expertise:

Inventory Model, Parking lot problems, Analytic Hierarchy Process, Optimization

I confirm that I have read this submission and believe that I have an appropriate level of expertise to confirm that it is of an acceptable scientific standard.

# Reviewer report: Reviewer response for version 2

**DOI:** 10.5256/f1000research.161819.r243837

**Published:** 2024-03-05

**Authors:** Mohammad Faisal Khan

**Affiliations:** 1 Saudi Electronic University, Riyadh, Riyadh Province, Saudi Arabia

Authors focused on an efficient and easy method for solving the given system of differential-algebraic equations (DAEs) of second order. The method includes computing the approximate solutions with rapidly and efficiently with the help of a semi-analytical method known as Adomian decomposition method (ADM). The logic of this method is simple and straightforward to understand. To demonstrate the proposed method, they presented several examples and the computations are compared with the exact solutions to show the efficient. They also claimed that one can employ this logic to different mathematical software tools such as Maple, SCILab, Mathematica, NCAlgebra, Matlab etc. for the problems in real life applications.

**General Comments:**

 The paper is organized in well-order and interesting to the general readers. The main text is to propose a method for DAEs using ADM which produces quick approximate solution which is novel as far as my knowledge. This paper can be *accepted*.

**Minor comments:**

1. In the paper, authors are focused on only second order DAEs. Is this technique applicable for higher order DAEs?
2. In the integral operator I, authors have taken lower value of the integral zero. What is the reason for fixed lower value zero?  If the lower value is not fixed, say c (constant), that are changes occur in the process?

Is the work clearly and accurately presented and does it cite the current literature?

Yes

If applicable, is the statistical analysis and its interpretation appropriate?

Yes

Are all the source data underlying the results available to ensure full reproducibility?

Yes

Is the study design appropriate and is the work technically sound?

Yes

Are the conclusions drawn adequately supported by the results?

Yes

Are sufficient details of methods and analysis provided to allow replication by others?

Yes

Reviewer Expertise:

Optimisation, Special functions,

I confirm that I have read this submission and believe that I have an appropriate level of expertise to confirm that it is of an acceptable scientific standard.

# Response

**Published:** 2024-03-06

**Authors:** Shanmugasundaram P

**Affiliations:** Department of Mathematics, Mizan-Tepi University, Mizan Teferi, Southern Nations, Nationalities, and People's Region, Ethiopia

The authors of this paper are very thankful to the reviewer for giving valuable comments on this paper. The response to the minor comments are as follows:

 1. In the paper, authors are focused on only second order DAEs. Is this technique applicable for higher order DAEs?

 Response: The proposed technique in this paper is applicable to the higher-order DAEs. For the sake of simplicity, we have taken 2-order DAEs.

 2. In the integral operator I, authors have taken lower value of the integral zero. What is the reason for a fixed lower value of zero?  If the lower value is not fixed, say c (constant), are changes occur in the process?

 Response: The lower value of the integral can be any number. Based on this, we compute integrals in the proposed algorithm. For simplicity, we took the lower value to be zero.
